# Requirements and Variability Affecting the Durability of Bonded Joints

**DOI:** 10.3390/ma13061468

**Published:** 2020-03-23

**Authors:** Rhys Jones, Daren Peng, John G. Michopoulos, Anthony J. Kinloch

**Affiliations:** 1 Centre of Expertise for Structural Mechanics, Department of Mechanical and Aerospace Engineering, Monash University, Clayton, VI 3800, Australia; daren.peng@monash.edu; 2 Computational Multiphysics Systems Laboratory, Code 6394, Center for Materials Physics and Technology, US Naval Research Laboratory, Washington, DC 20375, USA; john.michopoulos@nrl.navy.mil; 3 Department of Mechanical Engineering, Imperial College London, Exhibition Road, London SW7 2AZ, UK; a.kinloch@imperial.ac.uk

**Keywords:** CMH-17-3G, JSSG-2006, MIL-STD-1530D, PABST, A4EI, operational aircraft, variability in fatigue crack growth

## Abstract

This paper firstly reveals that when assessing if a bonded joint meets the certification requirements inherent in MIL-STD-1530D and the US Joint Services Standard JSSG2006 it is necessary to ensure that: (a) There is no yielding at all in the adhesive layer at 115% of design limit load (DLL), and (b) that the joint must be able to withstand design ultimate load (DUL). Secondly, it is revealed that fatigue crack growth in both nano-reinforced epoxies, and structural adhesives can be captured using the Hartman–Schijve crack growth equation, and that the scatter in crack growth in adhesives can be modelled by allowing for variability in the fatigue threshold. Thirdly, a methodology was established for estimating a valid upper-bound curve, for cohesive failure in the adhesive, which encompasses all the experimental data and provides a conservative fatigue crack growth curve. Finally, it is shown that this upper-bound curve can be used to (a) compare and characterise structural adhesives, (b) determine/assess a “no growth” design (if required), (c) assess if a disbond in an in-service aircraft will grow and (d) to design and life in-service adhesively-bonded joints in accordance with the slow-growth approach contained in the United States Air Force (USAF) certification standard MIL-STD-1530D.

## 1. Introduction

### 1.1. Background and Quasi-Static Design Considerations

The delaminations and disbonds that have been found in US Navy [1] and Royal Australian Air Force (RAAF) [2] F/A-18 inner wing lap splice joints (IWSLJ), have resulted in a re-examination of disbonding in adhesively bonded joints [3]. Much of the methodology currently used to design bonded joints in military aircraft is based on tools that were first validated as part of the USAF Primary Adhesively Bonded Structure Technology (PABST) program [4,5], specifically the computer program A4EI, which uses the strain energy density failure criteria [6] to assess cohesive failure in the adhesive. Indeed, CMH-17-3G [3] recommends using the computer program A4EI [5], which is an extension of the double lap joint solution given in [6], for the design of both bonded joints and bonded repairs to composite airframes. The ability of A4EI to accurately predict the quasi-static failure loads for simple joint configurations was first validated as part of the USAF Primary Adhesively Bonded Structure Program (PABST) [4]. Its ability to accurately predict the failure loads associated with axially-loaded complex metal to composite step lap joints was established in [7]. Indeed, CMH-17-3G states that A4EI can be used to design/assess bonded joints, and that this “avoids the need for elaborate finite element calculations.” Whilst CMH-17-3G did not give examples, [2] has shown that, for an axially-loaded bonded multi-step bonded lap joint, provided failure in the adhesive is cohesive, the A4EI computer code and finite element analyses do indeed yield similar estimates of the load carrying capacity.

One of the primary recommendations contained in [4] was that, to avoid durability issues, the adhesive should not be loaded beyond yield. This is an extension of the predictions for quasi-static failure loads in order to try to avoid durability problems. Thus, using this simplistic design approach of applying a “knock-down” factor to the quasi-static strength predictions, then no crack growth is permitted in the joint when in-service and it is assumed that durability problems will now be avoided. This recommendation is reflected in MIL-STD-1530D Section 5.2.4 [8], which states:

“Stress and strength analysis shall be conducted to substantiate that sufficient static strength is provided to react all design loading conditions without yielding, detrimental deformations and detrimental damage at design limit loads and without structural failure at design ultimate loads.”

The key phrase here is the statement that there be no yielding and detrimental damage at design limit load (DLL). There is a related statement in JSSG-2006 [9]. The difference being that the JSSG-2006 requirement is linked to 115% DLL. However, the current approach to designing bonded joints, as delineated in CMH17-3G [8], is to ensure that the strength of the joint is beneath design ultimate load. Unfortunately, as shown in Appendix A, it does not follow that, if a joint does not fail at design ultimate load (DUL), it will not yield at 115% DLL, or at 100% DLL. (The requirement that there be no yielding in the adhesive at DLL was a key design requirement in the Mirage III bonded doubler repair program [10,11], with the doublers being fitted to the RAAF fleet). As such, the way in which A4EI is commonly used does not necessarily ensure that a bonded joint will meet the guidelines given in the PABST report [4], or the certification requirements inherent in MIL-STD-1530D and JSSG-2006, see Appendix A for details. As such, it does not ensure that the adhesive bond will be durable. (It should be noted that discussions on how to design so as to achieve a durable joint are given in [12,13]). Consequently, as per the certification standard, the design/assessment of bonded joints should establish that:

1. The adhesive in the joint should not yield at 115% DLL;
2. The adhesive in the joint should not failure at DUL;
3. The joint should have an adequate fatigue life.

(Noting of course that, typically, DUL = 1.5 DLL [8,9])

### 1.2. Cyclic-Fatigue Design Considerations

**Figure 1:**
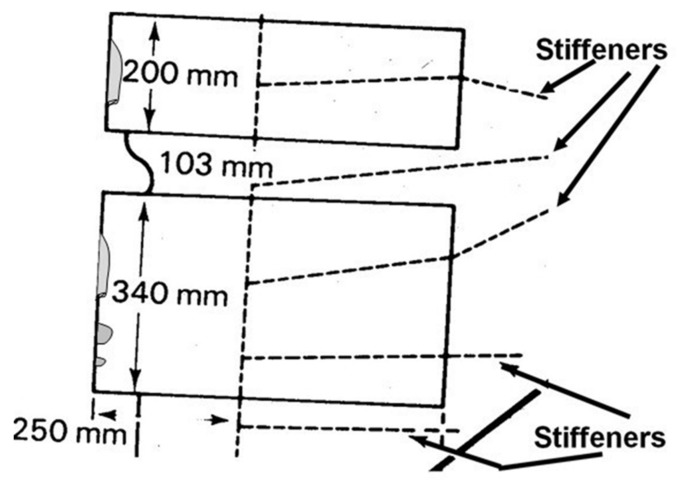
Schematic of outboard-edge disbonds (grey areas) at 923 air-flight hours in RAAF aircraft A15-5, from [15].

The belief that a bonded joint that meets quasi-static strength requirements will also meet durability requirements, based upon a “no crack growth” criterion from a “knock-down” factor applied to the quasi-static strength analysis, was invalidated by the failure of the boron-fibre epoxy composite doublers on the upper surface of F-111 aircraft [13,14]. Even though the airframe passed cold proof load (CPLT) tests at −40^o^ F, extensive delamination/disbonding arose shortly after undergoing CPLT [13,15] (i.e. in less than 100 flight hours). These delaminations/disbonds initiated at the edges of the doublers, see Figure 1. Examination of the doublers subsequently determined that whilst crack growth initiated in the adhesive it subsequently led to delamination in the boron/epoxy doubler [13,15]. A schematic diagram that illustrates the extent of one such disbond/delamination is shown in Figure 1. Here it should be noted that repair doublers were fitted to the RAAF fleet of twenty F-111C aircraft with a total of seventy-eight bonded doublers being fitted to the RAAF F-111 fleet. In some instances disbonds/delaminations arose between 729 and 1233 flight hours, after being fitted, and when found they tended to be fairly extensive, see Figure 1 [15].

The F-111 disbonds highlighted the importance of designing bonded joints so as to allow for the fatigue threshold(s), where the rate of fatigue crack growth attains a lower limit, associated with small naturally occurring initial flaws in the adhesive. The need to establish the inspection intervals associated with this repair also highlighted the need to be able to predict the slow growth of cracks/disbonds in the adhesive. The growth of small sub-millimetre cracks in bonded repairs/joints is not unique to the F-111 boron epoxy doubler. Cracking in adhesive bonds and subsequent delamination has also been observed in the doublers applied to Canadian CF-5 and AIRBUS A310 aircraft [16,17].

The need to account for the fatigue thresholds associated with such naturally occurring initial flaws, was also highlighted by Schoen, Nyman, Blom and Ansell [18], who stated:

“During certification of the AIRBUS A320 vertical fin, no delamination growth was detected during static loading. The following fatigue loading of the same component had to be interrupted due to large delamination growth. This demonstrates the importance of using the threshold value instead of the static value for delamination growth in the design of composite structures.”

Methods for determining the “worst case” thresholds necessary for a conservative design will be discussed later in this paper.

Since the majority of adhesively bonded structures only experience relatively low stress levels, there are relatively few reported instances of in-service disbond growth. Nevertheless, a range of examples can be found in [1,13,16,17, 19,20]. One instance, that involved both disbond and delamination growth in a US Navy aircraft, is discussed in [1]. In this instance disbonding was attributed to a problem with the surface treatment of the titanium inner adherend [1]. (Indeed, it is very well established [4,5,6,11,12] that the typical complex, and expensive, treatments used for aluminium- and titanium-alloys prior to adhesive bonding in the aerospace industry lead to adhesive/substrate interfaces which are very resistant to environmental attack by ingressing water and other hostile liquids. Therefore, invariably, any relatively poor durability of adhesively-bonded joints typically only arises from interfacial attack, and hence a loss of adhesion, when the treatment is inadequately undertaken).

**Figure 2:**
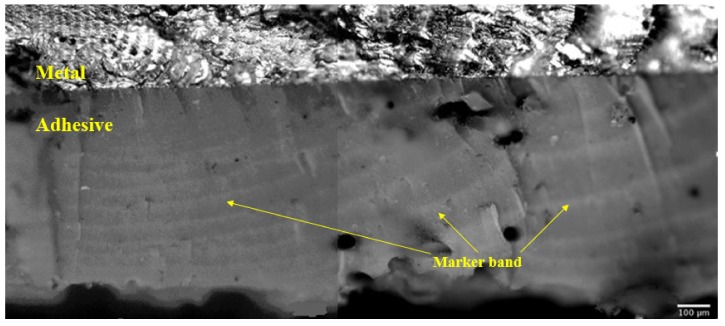
Illustration of (multiple) small crack growth in an adhesive in the tests discussed in [21], previously unpublished picture.

The growth of multiple small cracks in the structural film adhesive FM300K tested under marker block loading was also seen in [21]. These tests involved AA7050-T7351 specimens, which contained an array of small sub-mm cracks that were repaired using an externally bonded boron epoxy laminate, see Figure 2. In these tests, small cracks in the adhesive, that were less than approximately 0.01 mm in size, can be seen to have initiated and grown (in the adhesive) from the interface between the adhesive and the AA7050-T7451. Figure 2, when taken in together with fleet experience, and the examples given in Appendix B, suggests that the associated fatigue threshold for naturally occurring cracks in adhesives can be very low. This conclusion such mirrors the statements given in [22,23] and in Appendix X3 of ASTM E647-13a that for operational metallic structures the fatigue threshold is very low.

Let us now return to the certification requirement that there be no detrimental damage at 115% DLL. The Mirage III composite doubler program [11,12] and the PABST study [4], which were both undertaken in the mid to late 1970’s, recommended that the adhesive stresses should be kept beneath the fatigue limit of the adhesive. The PABST program [4], and the McDonnell Douglas study into bonded step lap joints [7], confirmed the statement given in [6] and in CMH-17-3G [3] that the performance of bonded joints was uniquely related to the strain energy density in the joint. The subsequent disbonding seen in the F-111 bonded doubler program, coupled with this realisation (i.e., that the performance of bonded joints was a function of the strain energy density in the adhesive) led to the realisation that there exists a limiting value of the strain energy density W_f_ that corresponds to the fatigue limit of the adhesive, and a simple method for determining W_f_ was developed [24]. This approach was first validated in [25,26,27]. It was also validated as part of an Airbus A330 full-scale fatigue test [26,28], and via a series of flight demonstrators on Boeing 727, 737 and 747 aircraft [29], as well as by a series of flight demonstrators on Boeing DC10 and MD11 and Lockheed L1011 civil transport aircraft [30].

In this context it should be noted that the PABST design methodology [4] recommended that to avoid fatigue damage the maximum shear stress in the adhesive should preferably be less than 50% of the yield stress. This conclusion is interesting since for two commonly used adhesives (i.e., Cytec FM73 and FM300), the value of W_f_ [12] corresponds (approximately) to 50% of the yield stress.

The above examples have shown that, for existing designs, from a sustainment perspective a slow growth approach to assessing disbond growth in operational aircraft can be essential. There would also appear to be a need, at the design stage, to allow for slow crack growth during the operational life of the airframe. This, in-turn, leads to the requirement for analysis tools for determining these inspection intervals. The 2009 US Federal Aviation Administration (FAA) Airworthiness Advisory Circular [23] was the first to allow approach to certifying adhesively-bonded structures. It has subsequently been adopted as part of the JSSG-2006 guidelines [9], as well as by MIL-STD-1530 [8]. New tools for performing this necessarily conservative design/analysis are discussed below when we now turn our attention to cyclic-fatigue crack growth in structural bonded joints. Design tools for assessing slow fatigue crack growth in adhesive joints are also discussed.

## 2. Materials and Methods

### 2.1. Research Methodology

The studies analysed in this paper have been either taken from peer reviewed journals that are publicly available, or from texts that are publicly available and their ISBN number has been quoted. Similarly, the references used in this paper were taken from: Peer reviewed journals that are publicly available, refereed Conferences and texts that are publicly available (in such cases) and their ISBN number has been quoted, or from Google searches. Of these references, fifty-five are in Journals listed in SCOPUS and WOS, three references are available on the US Department of Defense DTIC website (https://discover.dtic.mil/), one reference is on the NASA Technical Reports website, the Books/Book Chapters referenced are all listed in SCOPUS, one reference is contained in the Proceedings of ICM13 and the ISBN number is given, two reference can be found in ICCM22, etc. The exceptions to this were the Composite Materials Handbook CMH-17-3G [3], the USAF Certification documentation MIL-STD-1530D [8], and the USAF Joint Services Structural Guidelines JSSG2006 [9] all of which are unclassified and have no release restriction. Keywords used in these searchers were: Delamination growth, disbonding, durability, damage tolerance, Hartman–Schijve, disbond growth in operational aircraft, full-scale fatigue tests, aircraft certification, etc.

### 2.2. Theoretical Considerations

The paper by Lincoln and Melliere [31] presented the USAF approach to assessing the economic life of the USAF F-15 fleet. This paper was one of the first to illustrate how the tools needed for aircraft sustainment differ from those needed in design. The subsequent review paper [22] expanded on this, and explained that the fatigue crack growth equation needed for both design and sustainment can often be represented by the Hartman–Schijve, crack growth equation, which is a variant of the NASGRO equation, viz:

*da/dN* = *D*(Δκ)*^p^* (1)

Here *a* is the crack length/depth, *N* is the number of cycles, *D* and *p* are constants, and the crack driving force Δ*κ* is defined as per Schwalbe [32], viz:

Δ*κ* = (Δ*K* − Δ*K_thr_*) /(1 − (*K*_max_/A^*^))^1/2^ (2)

Here *K* is the stress intensity factor, $\Delta K=(K_{\max}-K_{\min})$ is the range of the stress intensity factor seen in a fatigue cycle, A^*^ is the cyclic fracture toughness, and Δ*K_thr_* is the fatigue threshold.

Whilst the origin of Equation (1) can be traced back to Hartman and Schijve [33], similar equations can be found in [34,35,36,37,38,39], and its link to fractal based crack growth equations is discussed in [40,41]. A range of examples showing how this formulation can be used to study aircraft sustainment related problems are given in [40,41,42,43,44,45,46,47,48,49]. Applications to bridge and rail steels are given in [50,51,52], to polymers in [53] and to bonded wooden joints in [54]. Equation (1) has also been shown to be able to represent the growth of both long and small cracks in additively-manufactured materials [55,56,57], to laser additive deposition (LAD) and cold spray repairs to metallic structures [58,59]. A feature of this formulation is that the scatter in the crack growth curves can often be accounted for by allowing for the variability in the threshold term Δ*K_thr_* [22,42,44,49,56,57].

For cohesive crack growth in adhesives many authors plot log *da/dN* as a function of either log Δ*G,* or as a function of log *G_max_*. The equivalent Hartman–Schijve equation can be obtained by replacing the terms Δ*K* and *K_max_* in Equation (2) by Δ*√G* and Δ*√G_max_*, respectively, so that we obtain [60]:

Δκ = (Δ√G − Δ√G_thr_) /(1 − √(G_max_/A))^1/2^ (3)

where the term *A* (≅ *G_c_*) is the apparent cyclic critical energy release rate, where *G_c_* is the adhesive fracture energy [60,61,62,63,64,65,66,67,68,69,70,71].

A key point raised in MIL-STD-1530D is the ability to characterise the variability in the fatigue thresholds and the associated disbond growth rates. Fortunately, [60,61,62] have shown that the variability in the delamination *da/dN* versus Δ*G* curves can be often captured by allowing for variability in the fatigue threshold (i.e. Δ*√G_thr_*) and the apparent fracture toughness, *G_c_*. As a result, it is now possible to obtain a statistically valid estimate of the variability in the fatigue thresholds and the associated disbond/delamination growth rates, see [60,61,62]. This approach is discussed in more detail in Section 3.

## 3. Characterising Cyclic Fatigue Crack Growth in Two F/A-18 Structural Epoxy Adhesives

The Mode II *da/dN* versus Δ*G_II_* curves for the thin film adhesives FM-300K and FM-300 (from Cytec, Woodland Park, NJ, USA) used in ‘F/A-18’ aircraft has been reported in [72]. The data reported in [72] was obtained using end-loaded split (ELS) test specimens tested at *R*-ratio’s of *R* = −1 and *R* = 0, and at test temperatures of 100, 20 and −50 °C. 

**Figure 3:**
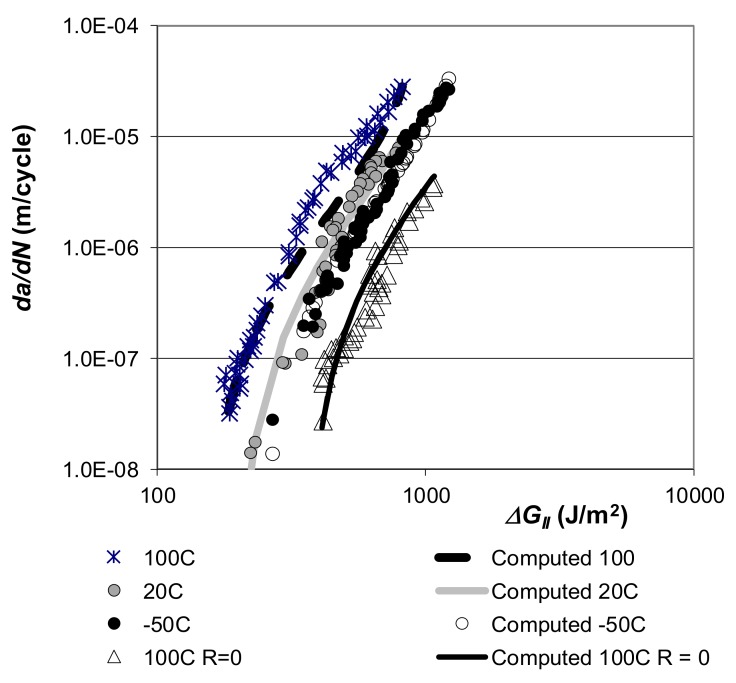
The measured [72] and computed [60] *R* = –1 and *R* = 0 *da/dN* versus $\Delta G_{II}$ curves for ‘‘FM300K”. (Where *da/dN* is the fatigue crack growth rate, with *a* being the crack length and *N* the number of fatigue cycles. The term *∆G* is the range of the applied strain-energy release-rate in the fatigue cycle, as defined by $\Delta G=G_{max}-G_{min}$; where $G_{max}\;\mathrm{and}\;G_{min}$ are the maximum and minimum values of the applied energy release-rates in the fatigue cycle, respectively).

**Figure 4:**
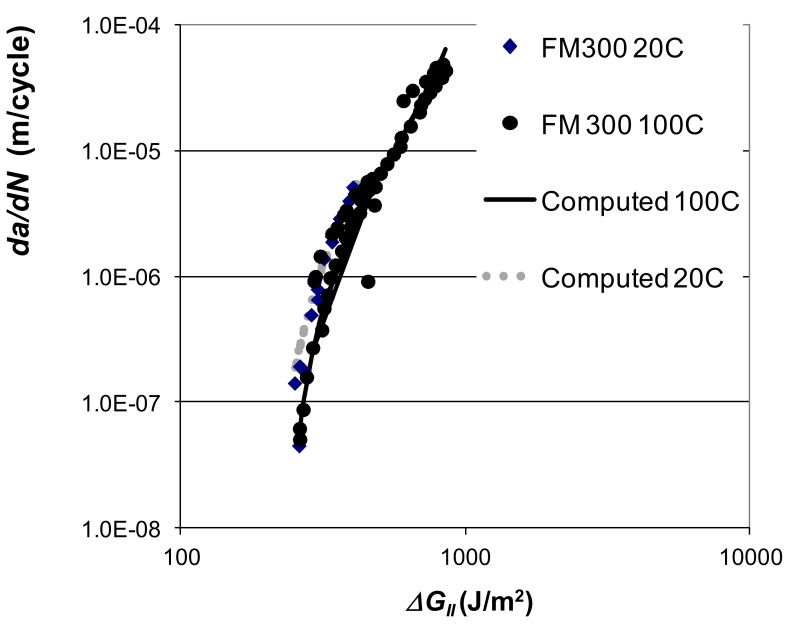
The measured [72] and computed [60] *R* = −1 *da/dN* versus $\Delta G_{II}$ curves for “FM300”.

**Figure 5:**
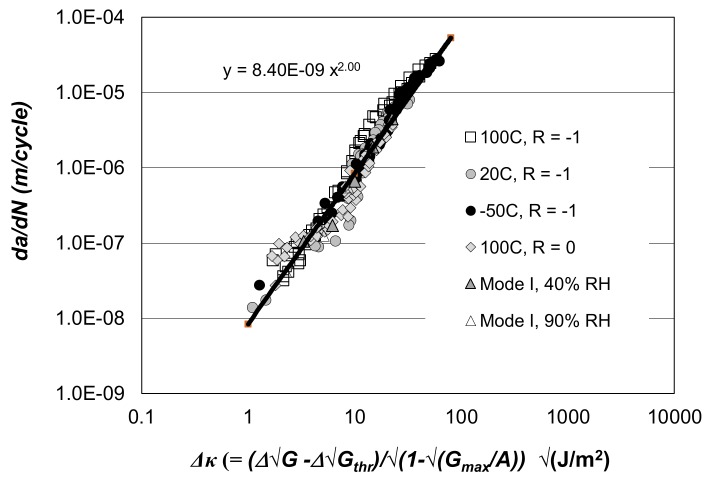
The *da/dN* versus Δκ relationship for Mode II and Mode I fatigue crack growth in “FM300K” [60]. (Mode II failure unless otherwise stated. Where $\Delta \sqrt{G}$ is the range of the applied strain-energy release-rate in the fatigue cycle, as defined by $\Delta \sqrt{G}=\sqrt{G_{max}}-\sqrt{G_{min}}$).

**Figure 6:**
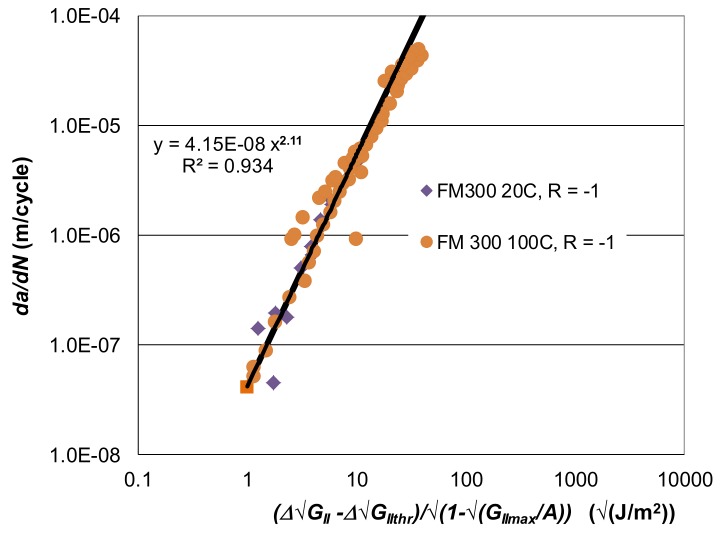
The *da/dN* versus Δκ relationship for Mode II fatigue crack growth in “FM300” [60].

The log *da/dN* versus log *∆G_II_* curves presented in [72] for these adhesives are shown in Figure 3 and Figure 4. Figure 5 and Figure 6, respectively, present the Mode II crack growth data shown in Figure 3 and Figure 4 replotted in accordance with Equation (1), with Δ*κ* as defined in Equation (3). Here we have plotted log *da/dN* against log Δκ, (Δκ = $\left[\frac{\Delta \sqrt{G_{II}}-\Delta \sqrt{G_{IIthr}}}{\surd \left\{1-\sqrt{G_{IImax}}/\sqrt{A}\right\}}\right]$). The values of the parameters used in Figure 5 and Figure 6, are given in Table 1 and Table 2. As shown in these tables, for a given adhesive system, the values of the constants D and p used are fixed. The values of the parameters *A* and $\Delta \sqrt{G_{IIthr}}$ have been derived from the individual experimental data. (It is of interest to note the differences in the values of *D* for the two different adhesives. However, the term *D* is essentially an empirical fitting factor, and cannot be directly related to the chemistry or the microstructure of the material). Figure 5 and Figure 6 reveal that, when Equations (1) and (3) are used to model crack growth, the *da/dN* versus Δκ relationship is essentially independent of *R*-ratio and temperature.

These plots reveal that Mode II crack growth behaviour in FM-300K and FM-300 may be represented using Equations (1) and (3). Indeed, when log *da/dN* is plotted against log Δκ, the effects of the *R*-ratio, and the test temperature collapse onto a single “master” (linear) curve with a correlation coefficient of 0.976, see Figure 5.

Equations (1) and (3), with the values of the parameters given in Table 1 and Table 2, were then used to compute the *da/dN* versus $\Delta G_{II}$ curves for these two epoxy adhesives. The resultant computed *da/dN* versus $\Delta G_{II}$ curves, for each test temperature and *R*-ratio, are also shown in Figure 3 and Figure 4. These figures reveal an excellent agreement between the measured and the computed *da/dN* versus $\Delta G_{II}$ curves. It would thus appear that the Mode II cyclic-fatigue behaviour of both “FM300K” and “FM300” may indeed be represented using Equations (1) and (3). Furthermore, for each adhesive the effects of both R-ratio and test temperature on crack growth are seen to collapse onto a single *da/dN* versus $\Delta G_{II}$ “master” curve, which exhibits a low degree of scatter. These “master” curves have a slope, *p*, of approximately two, see Figure 5 and Figure 6. The value of this exponent is relatively low in comparison to the values of about four to six for the linear regions for the measured data shown in Figure 3 and Figure 4.

### 3.1. Correlation of Mode I and Mode II Fatigue Crack Growth

**Figure 7:**
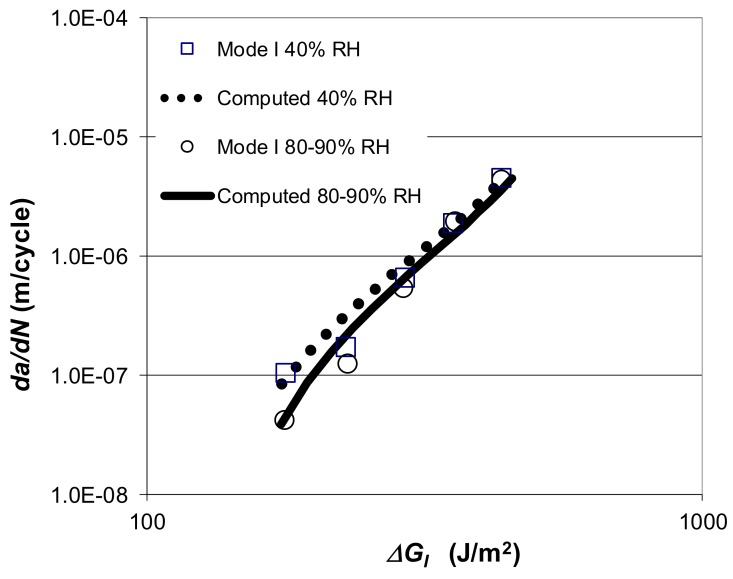
The measured [73] and computed [60] Mode I *da/dN* versus Δ*G_I_* for “FM300K”.

Let us next consider the *R* = −1 Mode I fatigue crack-growth data presented in [73], which was obtained using tapered double beam cantilever (TDCB) specimens, for crack growth in “FM300K”, for which the Mode II fatigue results are given above. These Mode I tests were conducted at 20 °C in both a 40% RH and a 90% RH environment. The measured *da/dN* versus *∆G_I_* plots [73] are shown in Figure 7. Figure 7 also contains, the curves computed using Equations (1) and (3) with the values of the constants *D* and *p* as per those used for the Mode II tests, see Table 1, and the values of the parameters *A* and *∆√G_thr_* as given in Table 3. The values of *A* and *∆√G_thr_* were calculated, as described above, with the value of *A* being a constant for these Mode I tests. As can be seen in Figure 7, there is excellent agreement between the experimental data and the Mode I curves computed using Equations (1) and (3).

The experimental Mode I data shown in Figure 7 is also shown replotted in Figure 5 according to the Equations (1) and (3), where log *da/dN* is plotted against log Δ*κ*, (Δκ = $\left[\frac{\Delta \sqrt{G_{I}}-\Delta \sqrt{G_{Ithr}}}{\surd \left\{1-\sqrt{G_{Imax}}/\sqrt{A}\right\}}\right]$). The values of the various parameters employed in these representations are given in Table 3. It should be noted that the master curve shown in Figure 6 for the Mode I crack growth curves is identical to that previously obtained to describe the Mode II fatigue data. Thus, for the adhesive FM300K the Hartman–Schijve approach appears to yield a convenient “master” curve relationship that describes both Mode I and the Mode II fatigue crack growth. Comparing the *da/dN* versus *∆G* plots for Mode II and Mode I crack growth, see Figure 3 and Figure 7, respectively, it appears that at 20 °C the fatigue behaviour of the epoxy-film adhesive “FM300K” is significantly superior under Mode II loading. This is reflected in the values of the terms *A* and *∆√G_thr_* used in Equation (3) being significantly greater for tests conducted under Mode II than under Mode I fatigue loading, see Table 1 and Table 3.

### 3.2. Characterising the Variability in Fatigue Crack Growth in Structural Adhesives

**Figure 8:**
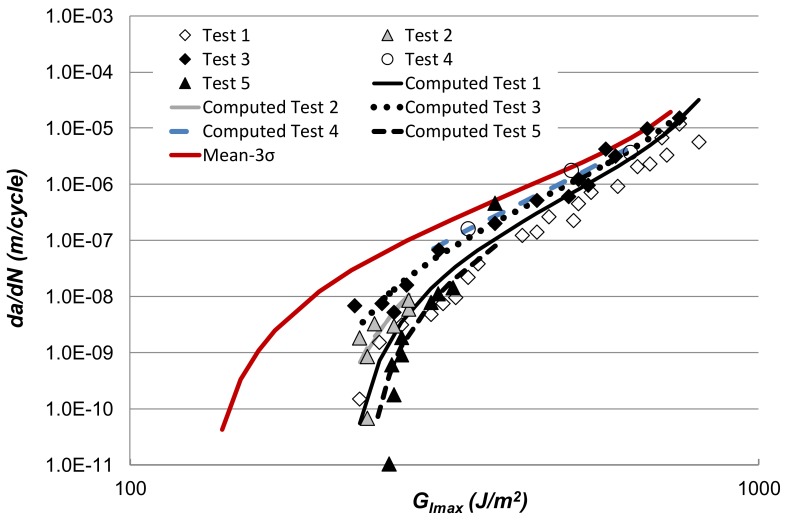
The measured [74] and computed Mode I *da/dN* versus *G_Imax_* curves for the rubber-toughened epoxy-film adhesive (“EA9628”). (The subscript I refers to Mode I (tensile-opening loading).

Let us next address how to characterise the variability of the crack growth under fatigue loading in structural adhesives. Several examples that illustrate the ability of the Hartman and Schijve equation to represent crack growth in structural adhesives were given in [60]. One such example involved fatigue crack growth data [74] for the rubber-toughened epoxy adhesive “EA9628” (Hysol Dexter, Irvine, CA, USA) obtained using double cantilever beam (DCB) tests performed at *R* = 0.5, 23 ± 1 °C and a relative humidity of 55% ± 5% RH. A plot of the experimentally obtained log *da/dN* versus log *G_Imax_* data is given in Figure 8. This figure clearly illustrates the variability that can arise in crack growth in adhesives. As previously noted, the ability to capture this variability, and to determine statistically valid “worst case” crack growth rates is essential for the certification/assessment of bonded airframes. Fortunately, as has been mentioned above, the Hartman and Schijve equation has the ability to capture this variability by allowing for variability in the fatigue threshold. This is aptly illustrated in Figure 8, which presents both the measured and computed curves. Here the computed *da/dN* versus *G_Imax_* curves were determined using Equation (1), with Δ*κ* as defined in Equation (3), with the values of *D*, *A* and *p* all kept fixed. The variability in the curves was captured by allowing the threshold term Δ√*G_Ithr_* to vary between 7.65 to 6.50 √(J/m^2^), see Table 4. This now enables an estimate of the “mean–3σ” value of Δ√*G_Ithr_* to be estimated. This in-turn enables an estimate of the “mean–3σ” upper-bound (i.e., worst case) *da/dN* versus *G_Imax_* curve to be computed, see Figure 8.

Thus, a methodology has been established for estimating a valid upper-bound curve which encompasses all the experimental data and provides a conservative fatigue crack growth curve which is representative of the structural adhesive. Such a valid, upper-bound curve can then employed for: (a) The characterisation and comparison of adhesives, (b) a “no growth” design (if required), (c) for assessing if a disbond in an in-service aircraft will grow, and (d) the design and lifing of in-service adhesively-bonded aircraft structures where material allowable properties have to be inputted into a disbond slow growth analysis for cyclic-fatigue loading.

To further illustrate the above, let us consider the *R* = 0.1 *da/dN* versus Δ*K* data presented in [75] for a toughened-epoxy structural adhesive tested at room temperature at a frequency of 20 Hz, and a *R* = 0.1. In this context, [60] revealed that the variability in the various replicate tests performed in [75] could also be captured using the Hartman and Schijve equation, and allowing for variability in the fatigue threshold.

## 4. Cyclic Fatigue Crack Growth in Nano-Composite Adhesives

**Figure 9:**
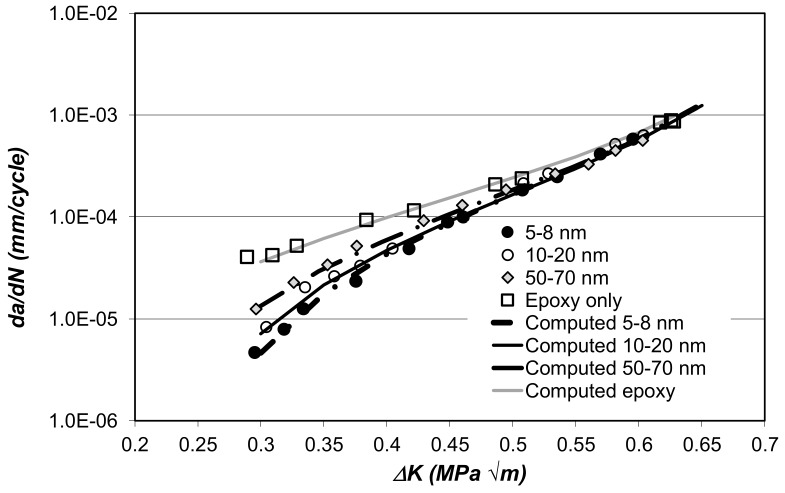
The measured and computed *da/dN* versus Δ*K* curves for the MWCNT nano-composite studied in [80], from [69].

The problems associated with disbond and delamination growth in current airframes, which employ thermosetting polymers as structural adhesives and/or matrices for fibre-reinforced composite materials, has led to a focus on the potential use of polymeric nano-composites [69,76,77,78,79,80]; and the full details of the chemistry and cure schedules of the nano-composite epoxy materials are given in [79,80]. The ability of the Hartman–Schijve equation to capture delamination growth in such nano-composites was first documented in [69]. This study revealed that, just as the variability in the delamination growth curves in traditional adhesives could be captured by allowing for changes in the threshold and fracture toughness terms in the Hartman–Schijve equation, the improvements in the damage tolerance associated with nano-composites could be captured in the same fashion. To this end it was shown [69] that the *da/dN* versus Δ*K* curves presented in [80] for a multi-walled carbon-nanotube reinforced epoxy nano-composite (MWCNT), where the MWCNT’s had several different diameters, namely 5–8, 10–20 and 50–70 nm, could be captured using Equation (1), with Δ*κ* as defined in Equation (2), with the values of Δ*K_thr_*, *A*, *D* and *p* as given in Table 5, see Figure 9, where $\Delta K_{thr}=K_{\mathrm{thr}.\max}-K_{\mathrm{thr}.\min}$.

**Figure 10:**
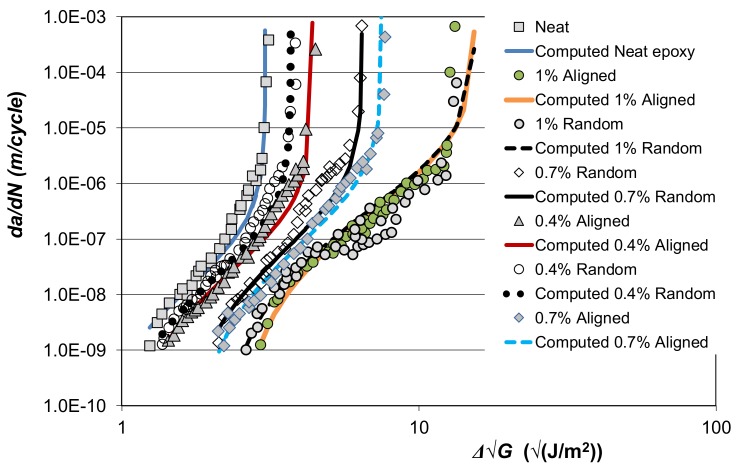
Comparison of the measured [79] and computed *da/dN* versus $\Delta \sqrt{G}$ curves in the various nano-composite adhesives.

To continue this study let us consider the results presented in [79] on the effects of different concentrations of carbon nano-fibres (CNFs) on crack growth in a composite DCB. The CNFs had diameters that ranged from approximately 70 to 300 nm, and lengths in the range of 30 to 200 mm, and DCB tests were performed on samples that had 0.4, 0.7 and 1.0 wt.% of CNFs. The composite adherends consisted of 12 plies of unidirectional T700 carbon fibre/epoxy pre-preg, see [79] for more details. Reference [79] presented the measured delamination growth rate, *da/dN*, plotted as a function of Δ√*G* (= √*G_max_* − √*G_min_*), see Figure 10. The corresponding computed curves, which were determined using Equations (1) and (3), are also shown in Figure 10. The values of *D*, *p*, *A* and Δ√*G_thr_* used in Figure 10 are given in Table 6. Here we again see that the effect of the nano-constituents on disbond growth can be captured by allowing for changes in the threshold and fracture toughness terms in Equation (3). (A similar situation arises for additively-manufactured materials, where the variability in the *da/dN* versus Δ*K* curves associated with variations in the manufacturing processes is captured by allowing for variability in the threshold and fracture toughness terms in Equation (2)).

One reason for showing these two particular examples is that:

1. In one case the addition of the nano-constituent only affected the fatigue threshold.
2. In the other case the addition of the nano-constituent affected both the fatigue threshold and the fracture toughness.

This suggests that the effect of nano-constituents on the fatigue threshold and the toughness of the adhesive are not strongly linked (i.e., are relatively independent of each other).

Unfortunately, most studies into delamination/disbond growth in nano-composites and adhesives only present the results of a relatively few replicate (i.e., repeat) tests. Consequently, to meet the requirements inherent in MIL-STD-1530D, further studies with sufficient repeat testing to characterise the effects of material, processing, and manufacturing variability are needed. Such tests would then enable the “mean-3σ” upper-bound curves to be obtained.

## 5. Conclusions

This study has shown that:

1. When assessing if a bonded joint meets the certification requirements inherent in MIL-STD-1530D and the US Joint Services Standard JSSG2006, it is necessary to ensure that (a) there is no yielding in the adhesive layer at 115% of design limit load, and (b) that the joint must be able to withstand design ultimate load.
2. However, the belief that a bonded joint that meets quasi-static strength requirements will also meet durability requirements, based upon a “no crack growth” criterion from a “knock-down” factor applied to the quasi-static strength analysis, has been shown to be invalid in the applications studied.
3. Indeed, considering applications of structural adhesive bonding in aircraft has highlighted the importance of designing bonded joints so as to allow for the fatigue threshold(s), where the rate of fatigue crack growth attains a lower limit, associated with small naturally occurring initial flaws in the adhesive. The requirement to establish the necessary inspection intervals associated with any bonded repairs also highlights the need to be able to predict the slow growth of cracks/disbonds in the adhesive.
4. Fatigue crack growth in structural adhesives and in nano-reinforced epoxies under cyclic fatigue loading can be modelled Equations (2) and (3).
5. For the structural adhesives FM300 and FM300K the fatigue data from both Mode I and Mode II loading collapse onto essentially the same *da/dN* versus Δκ master curve, regardless of the *R*-ratio and test temperatures that ranged from 20 to 100 °C.
6. It would appear that the variability in the fatigue crack growth data, that is typically observed in adhesives, can be captured by allowing for the variability in the fatigue threshold term in the crack driving force Δ*κ* is defined as per Schwalbe [32] (i.e., Equation (3)).
7. Thus, a methodology has been established for estimating a valid upper-bound curve, for cohesive failure in the adhesive, which encompasses all the experimental data and provides a conservative fatigue crack growth curve which is representative of the structural adhesive. Such a valid, upper-bound curve can then employed for (a) the characterisation and comparison of adhesives, (b) a “no growth” design (if required), (c) for assessing if a disbond, that is found in an in-service aircraft, will grow and (d) the design and lifing of in-service adhesively-bonded aircraft structures, where material allowable properties have to be inputted into a disbond slow growth analysis for cyclic-fatigue loading.

## Figures and Tables

**Table 1:** Values of the parameters used in [60] to represent Mode II crack growth in “FM300K”.

Test	*D*	*p*	*A* (J/m^2^)	$\Delta \sqrt{G_{IIthr}}(\surd (J/m^{2}))$
100 °C and *R* = −1	8.40 × 10^−9^	2.00	975	12.5
20 °C and *R* = −1	8.40 × 10^−9^	2.00	1200	14.1
−50 °C and *R* = −1	8.40 × 10^−9^	2.00	1500	15.5
100 °C and *R* = 0	8.40 × 10^−9^	2.00	2700	10.0

Note: *D* and *p* are constants; *A* is the apparent cyclic critical energy release rate (≅ *G_c_*); $\Delta \sqrt{G_{thr}}$ is the range of the fatigue threshold value of $\Delta \sqrt{G}$ as defined below: $\Delta \sqrt{G_{thr}}\;=\sqrt{G_{thr.max}}-\sqrt{G_{thr.min}}$. The subscript II indicates Mode II (in-plane shear) loading R is the displacement, or load, ratio.

**Table 2:** Values of the parameters used in [60] to represent Mode II crack growth in “FM300”.

Test	*D*	*p*	*A* (J/m^2^)	$\Delta \sqrt{G_{IIthr}}(\surd (J/m^{2}))$
100 °C and *R* = −1	4.15 × 10^−8^	2.11	1100	15.3
20 °C and *R* = −1	4.15 × 10^−8^	2.11	755	15.0

**Table 3:** Values of the parameters employed in the representation of Mode I crack growth in “FM300K”, from [60].

Test Relative Humidity (RH)	*D*	*p*	*A* (J/m^2^)	$\Delta \sqrt{G_{Ithr}}(\surd (J/m^{2}))$
40% RH	8.40 × 10^−9^	2.00	630	9.8
80%–90% RH	8.40 × 10^−9^	2.00	630	10.5

**Table 4:** Values of the constants employed to compute the *da/dN* versus ***G_Imax_*** relationships shown in Figure 8.

Test	*D*	*p*	*A* (J/m^2^)	$\Delta \sqrt{G_{Ithr}}(\surd (J/m^{2}))$
1	2.07 × 10^−9^	2.87	900	7.42
2	2.07 × 10^−9^	2.87	900	7.14
3	2.07 × 10^−9^	2.87	900	6.80
4	2.07 × 10^−9^	2.87	900	6.50
5	2.07 × 10^−9^	2.87	900	7.65
Mean-3σ	2.07 × 10^−9^	2.87	900	5.72

**Table 5:** Values of the constants employed to compute the *da/dN* versus Δ*K* curves shown in Figure 9.

Type of Nano-Composite	*D*	*p*	$A^{*}\;(\mathrm{MPa}\surd m)$	$\Delta K_{thr}\;\left(\mathrm{MPam}\right)\;$
5–8 nm diameter MWCNT	8.0 × 10^−7^	2.0	0.8	0.24
10–20 nm diameter MWCNT	6.5 × 10^−7^	2.0	0.8	0.22
50–70 nm diameter MWCNT	5.5 × 10^−7^	2.0	0.8	0.18
Neat epoxy, no MWNT	4.0 × 10^−7^	2.0	0.8	0.07

**Table 6:** Values of the constants used to represent the *da/dN* versus $\Delta \sqrt{G}$ curves given in [79].

Type of Nano-Composite	*D*	*p*	*A* (J/m^2^)	$\Delta \sqrt{G_{thr}}(\surd (J/m^{2}))$
Neat epoxy	6.0 × 10^−9^	2.2	37	0.72
0.4% Random	6.0 × 10^−9^	2.2	55	0.89
0.4% Aligned	6.0 × 10^−9^	2.2	77	1.0
0.7% Random	6.0 × 10^−9^	2.2	160	1.58
0.7% Aligned	6.0 × 10^−9^	2.2	225	1.76
1% Random	6.0 × 10^−9^	2.2	960	2.19
1% Aligned	6.0 × 10^−9^	2.2	950	2.49

**Figure A1:**
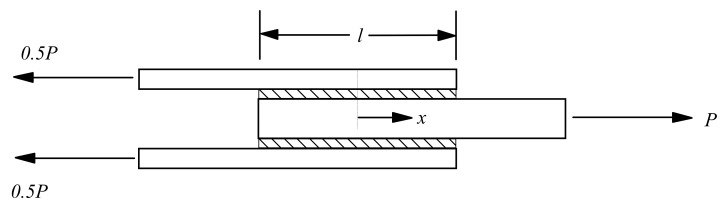
Geometry and notation for adhesive bonded double lap joint.

**Figure A2:**
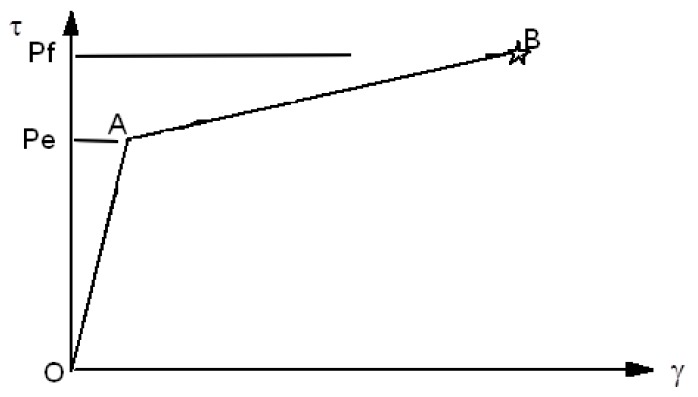
Schematic diagram of the adhesive shear stress versus shear strain relationship.

**Figure A3:**
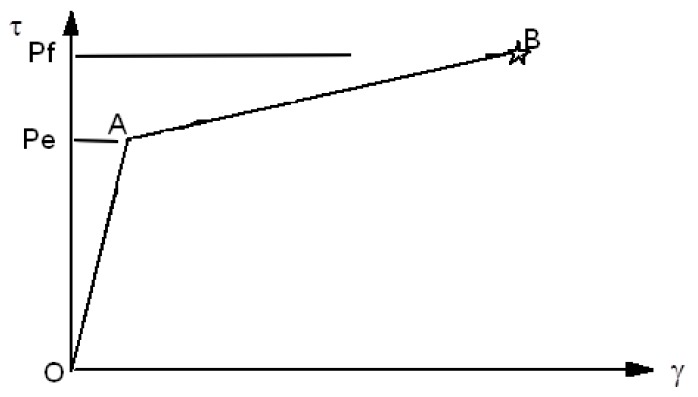
Schematic diagram of the adhesive shear stress versus shear strain relationship.

**Figure A4:**
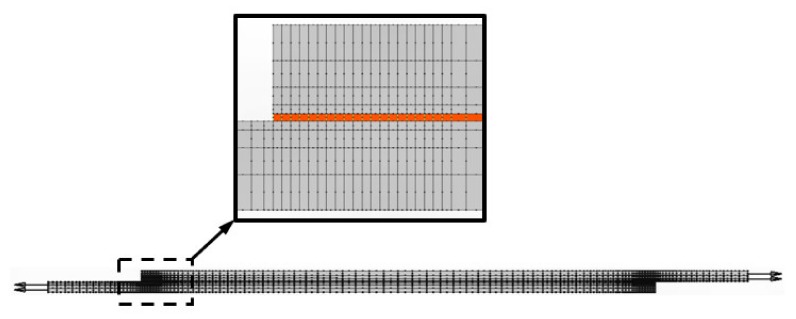
Finite element model of the joint.

**Figure A5:**
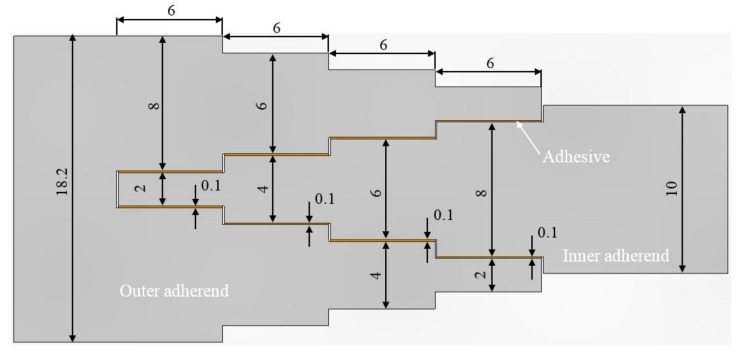
Schematic diagram of the cross-section of the four-step bonded joint studied in [2], dimensions are in mm.

**Figure A6:**
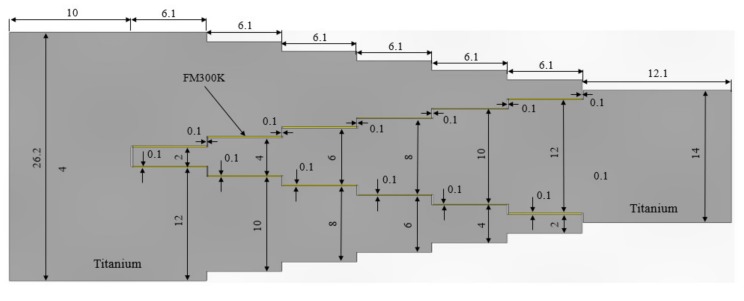
Schematic diagram of the cross-section of the six-step bonded joint, dimensions are in mm.

**Figure A7:**
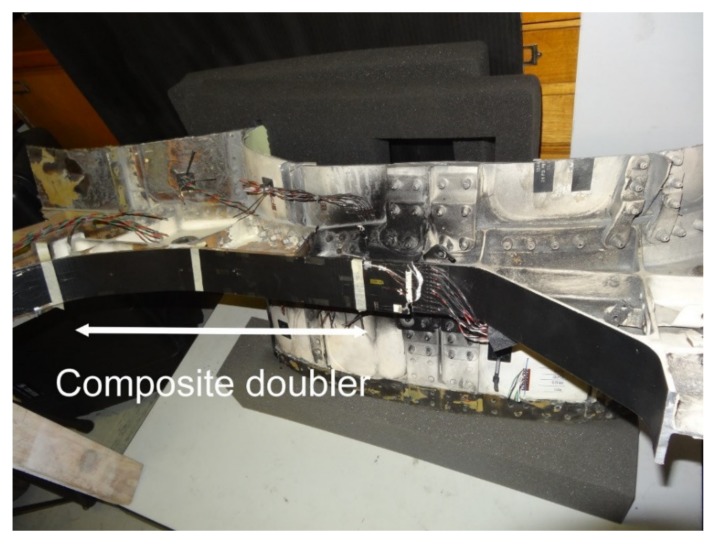
Overall view of a section of the Y488 bulkhead, with the doubler and surrounding section painted black prior to performing lock-in thermography [83].

**Figure A8:**
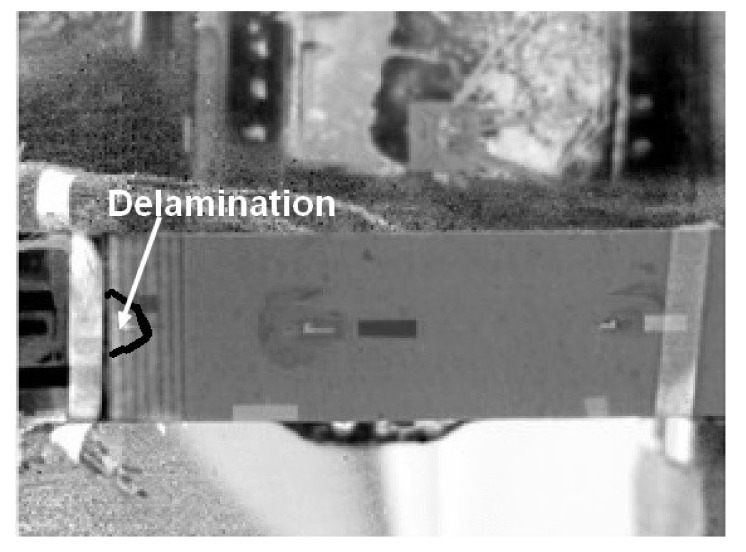
Schematic diagram showing the location and extent of the disbond [83].

## Appendix A. Counter Examples

*Consider the hypothesis:*

Hypothesis: Designing a bonded joint such that the joint will not fail at the ultimate design load will ensure that there will be no yielding at 115% DLL. (This requirement is as per JSSG-2006 [9]).

Definitions: Consider the simple double lap joint shown in Figure A1, where the adhesive has a shear stress, τ, versus shear strain, γ, relationship as given by the curve OAB in Figure A2. Point A in Figure A2 corresponds to the yield point of the adhesive and point B corresponds to failure of the adhesive. The loads at points A and B are defined by Pe and Pf, respectively, and We and Wc are the areas under the curves OA and OAB, respectively.

Counter Example 1: The A4EI user manual example

Given that a hypothesis can be disproven via a single counter example, let us first consider the example given in the A4EI user manual [5] which was used to illustrate how to determine the failure load of a simple double lap joint. The geometry of this joint, which consisted of a 3.048 mm thick inner aluminium alloy adherend bonded to two 1.524 mm thick outer aluminium-alloy adherends using a 0.127 mm thick layer of FM73, is shown in Figure A3.

The material properties used in [5] were:

Adhesive: G = 344.7 MPa, τ_y_ = 34.5 MPa, ϒ_max_ = 0.5, ν_adhesive_ = 0.3;

Aluminium adherends: E = 68,947 MPa, ν_adherend_ = 0.31

where G is the shear modulus of the adhesive, τ_y_ is the shear yield stress of the adhesive, ϒ_max_ is the maximum shear strain of the adhesive, ν_adhesive_ is the Poisson’s ratio of the adhesive, E is the tensile modulus of the adherend and ν_adherend_ is the Poisson’s ratio of the adherend.

As in [5], the adhesive was assumed to exhibit an elastic perfectly plastic response. A non-linear finite element analysis of this joint configuration was performed and because of symmetry only half of the joint was analysed, see Figure A4. (To check convergence the analysis was also run with double the number of nodes and elements. The results differed by less than 1%. Also, it should be noted that the AE4I approach ignores what [81,82] termed “artificial singularities” that arise at the ends of the joint due to the two dissimilar materials, i.e. the adhesive and the adherend). The failure loads computed using finite element analysis and A4EI are compared in Table A1, where we see excellent agreement.

**Table A1:** Predicted failure loads (N/mm) from the finite element (FE) and analytical (AE4I) methods.

Failure load	FE	A4EI	% Difference
Failure load (N/mm)	1791	1821	1.7

Firstly, for this joint configuration the failure load is predicted to be 1821 and 1791 N/mm via the A4EI and FE methodologies, respectively. Secondly, for this joint, A4EI gave the load to reach W_e_ (i.e., the yield point of the adhesive) to be approximately 607 N/mm. FE analysis returned a load of approximately 604 N/mm (i.e., a difference of less than 1%). Now, JSSG-2006 requires that there be no yielding in the adhesive at 115% design limit load (DLL). Thus, let us assume that the load to reach yielding at a value of W_e_ is equivalent to 115% DLL. It then follows that the predicted load on the joint at 150% DLL (i.e., at DUL) would be 792 N/mm. This load is significantly less than the actual failure load of the joint. Indeed, even if the MIL-STD-1530D requirements are followed (i.e., that no yielding of the adhesive occurs at 100% DLL), then the predicted failure load at 150% DLL would still only be 911 N/mm. Obviously, these calculated loads at DUL are significantly less than the predicted failure load of the joint, see Table A1. Hence, (a) the full load bearing capacity of the joint is far from being completely utilised from following either the JSSG-2006 or the CMH17-3G guidelines, and (b) conversely, as commented in Section 1, it does not follow that, if a joint does not fail at DUL, it will not yield at 115% DLL, or even at 100% DLL.

Counter Example 2: A four step lap joint under uniaxial axial loads

Let us next consider the four step lap joint studied in [2]. The joint was taken to be 60 mm wide and the geometry of the joint is as shown in Figure A5. As per [2] the adherends were taken to be Ti-6Al-4V, and the adhesives was assumed to be FM300K. The mechanical properties of the titanium adherends and the FM300K adhesive were taken to be as given in [2], viz:

Adhesive (FM300K): G = 393 MPa, shear yield stress = 39 MPa, maximum shear strain = 0.31, ν_adhesive_ = 0.3;

Adherend (Titanium): E = 110,316 MPa, ν_adherend_ = 0.3

This joint was considered to be subjected to uniaxial loading. A4EI gave an axial load of 1048 N/mm to reach W_e_. An elastic finite element analysis of the joint was also performed. This analysis revealed gave a load to W_e_ that differed from that computed using A4EI by less than 2%. Thus, assuming that the load to reach W_e_ occurs at 115% DLL, it follows that the calculated failure load at 150% DLL (i.e., at DUL) would be 1367 N/mm. On the other hand, A4EI gave a predicted failure load of 1872 N/mm. Consequently, the load at 150% DLL is again significantly less than the load bearing capacity of the joint.

Counter Example 3: A six-step lap joint under uniaxial axial loads

Let us next consider the 60 mm wide six-step lap joint studied in Figure A6. The adherends were taken to be Ti-6Al-4V and the adhesive was assumed to be FM300K. The mechanical properties of the titanium adherends and the FM300K adhesive were taken to be as in Counter Example 2. A4EI gave an axial load of 1432 N/mm to reach W_e_. An elastic finite element analysis of the joint was also performed. This analysis gave a load to reach W_e_ that differed from that computed using A4EI by less than 1%. Thus, assuming that the load to reach W_e_ occurs at 115% DLL, it follows that the calculated load at 150% DLL (i.e., at DUL) would be 1868 N/mm. However, A4EI gave a predicted failure load of 2621 N/mm. Consequently, the load at 150% DLL is yet again significantly less than the load bearing capacity of the joint.

Summary:

These three examples reveal, firstly, that the full load bearing capacity of the joint is far from being completely utilised from following either the JSSG-2006 or the CMH17-3G guidelines. Secondly, conversely, if the value of the DUL (i.e., for failure and as predicted using A4EI) is assumed to correspond to 150% DLL, then it follows that the load corresponding to either 115% DLL, or 100% DLL, would be significantly greater than the load for yielding of the adhesive, as given from the value of We which corresponds to the yield point of the adhesive. Hence, the adhesive will have undergone plastic yielding by the time the applied load reaches 115% DLL, or even 100% DLL. Thus, the hypothesis that designing a bonded joint such that the joint will not fail at the DUL will ensure that there will be no yielding at 115% DLL, or even 100% DLL, is disproven.

Consequently, as per the PABST recommendation and JSSG2006, the design/assessment of bonded joints should establish that:

1. The adhesive in the joint should not yield at 115% DLL;
2. There should be no failure at design ultimate load (DUL);
3. The joint should have an adequate fatigue life.

This conclusion is supported by fleet experience associated with the disbonding of the composite doublers on F-111 aircraft in service with the RAAF [13]. Here, despite passing cold proof load (CPLT) testing at −40^o^ F, there was subsequently extensive delamination/disbonding in under 1000 flight hours (at −40^o^ F the adhesive is more brittle and, hence, the failure strain of the adhesive is reduced) [13].

## Appendix B. Examples of Disbond Growth under Operational Loads

### Appendix B.1. Debonding of a Composite Repair to F-111 Lower Wing-Skin

In the early 1990s a 48 mm long crack was discovered in the wing-skin of RAAF F-111C aircraft A15-5. This crack was repaired using a bonded composite doubler. The doubler was designed in accordance with the guidelines outlined in [20]. When repaired the wing had seen approximately 4749.5 flight hours. However, small disbonds, which were less than 3 mm in diameter, were detected shortly after the wing had been repaired. By 5607 flight hours there were two significant disbonds. One had an edge length of approximately 57 mm, and a depth of approximately 19 mm. The other disbond had an edge length of approximately 48 mm, and a depth of approximately 10 mm, see [20].

The wing was; thus, retired from service and taken to the Defence Science and Technology Group for fatigue testing under a representative flight load spectra. After approximately 10,525 simulated flight hours it became apparent that these disbonds had grown extensively, and that new disbonds had arisen, see [20].

### Appendix B.2. Delamination in a Full-Scale Fatigue Tests on a Finnish Aircraft F/A-18 Boron-Fibre/Epoxy-Matrix Doubler Repair

Reference [83] outlined the findings of a full-scale fatigue test on a F/A-18 centre-barrel performed by the Australian Defence Science Group for the Finnish Air Force in order to evaluate the effect of a boron-fibre/epoxy doubler on the fatigue life of the aircraft. The doubler consisted of a 1.05 mm thick uni-directional laminate that was located, as shown in Figure A7, on the underside of the Y488 bulkhead. The doubler was approximately 403 mm long and 58 mm wide. Whilst the doubler itself withstood 6498 simulated flight hours, a disbond developed, see Figure A8, and grew during testing. This disbond appeared to initiate at the very start of the test.
